# Deregulated expression of polycomb repressive complex 2 target genes in a NF1 patient with microdeletion generating the *RNF135-SUZ12* chimeric gene

**DOI:** 10.1007/s10048-023-00718-8

**Published:** 2023-05-05

**Authors:** Viviana Tritto, Federico Grilli, Donatella Milani, Paola Riva

**Affiliations:** 1 Dipartimento di Biotecnologie Mediche e Medicina Traslazionale, Università degli Studi di Milano, Via Fratelli Cervi 93, 20054 Segrate, Italy; 2 Dipartimento Donna-Bambino-Neonato, UOSD Pediatria ad Alta Intensità di Cura, Fondazione IRCCS Ca’ Granda Ospedale Maggiore Policlinico, Via della Commenda 9, 20122 Milan, Italy

**Keywords:** Neurofibromatosis type I microdeletion syndrome, *RNF135-SUZ12* chimeric gene, Polycomb repressive complex 2, Early-onset neurofibromas, Gene expression regulation

## Abstract

Neurofibromatosis type I (NF1) microdeletion syndrome, accounting for 5–11% of NF1 patients, is caused by the heterozygous deletion of *NF1* and a variable number of flanking genes in the 17q11.2 region. This syndrome is characterized by more severe symptoms than those shown by patients with intragenic *NF1* mutation and by variable expressivity, which is not fully explained by the haploinsufficiency of the genes included in the deletions. We here reevaluate an 8-year-old NF1 patient, who carries an atypical deletion generating the *RNF135-SUZ12* chimeric gene, previously described when he was 3 years old. As the patient has developed multiple cutaneous/subcutaneous neurofibromas over the past 5 years, we hypothesized a role of *RNF135-SUZ12* chimeric gene in the onset of the patient’s tumor phenotype. Interestingly, *SUZ12* is generally lost or disrupted in NF1 microdeletion syndrome and frequently associated to cancer as *RNF135.* Expression analysis confirmed the presence of the chimeric gene transcript and revealed hypo-expression of five out of the seven analyzed target genes of the polycomb repressive complex 2 (PRC2), to which SUZ12 belongs, in the patient’s peripheral blood, indicating a higher transcriptional repression activity mediated by PRC2. Furthermore, decreased expression of tumor suppressor gene *TP53*, which is targeted by RNF135, was detected. These results suggest that RNF135-SUZ12 chimera may acquire a gain of function, compared with SUZ12 wild type in the PRC2 complex, and a loss of function relative to RNF135 wild type. Both events may have a role in the early onset of the patient’s neurofibromas.

**Supplementary Information:**

The online version contains supplementary material available at 10.1007/s10048-023-00718-8.

## Introduction

NF1 microdeletion syndrome (MIM#613675) is a clinical condition accounting for 5–11% of NF1 patients [1], caused by large deletions including *NF1* gene and frequently associated with a severe manifestation of neurofibromatosis type 1 [2]. On the basis of their recurrence and breakpoint location, the deletions are classified as types 1, 2, and 3, and atypical. The most represented NF1 deletion is type 1 (70–80%) [3], resulting from interchromosomal non-allelic homologous recombination during maternal meiosis [4, 5], occurring between the low-copy repeats NF1-REPa and NF1-REPc. The frequency of atypical deletions is around 8–10% of all NF1 microdeletions. They are heterogeneous not only for their different localization of breakpoints, but also for the mechanisms underlying the chromosomal rearrangements: non-allelic homologous recombination, DNA double-strand break repair, aberrant replication, and retrotransposon-mediated mechanisms [6]. The precise characterization of atypical NF1 deletions, at both genetic and clinical levels, can improve genotype-phenotype correlation in NF1 microdeletion syndrome. In fact, deletion of a subset of the 14 encoding genes, included in the most frequent type 1 microdeletion, or the presence of specific flanking genes, could reveal the role of a specific gene in the onset of specific clinical signs. Here, we report on a young patient with NF1 microdeletion syndrome, previously described when he was 3 years old [7]. He carries an atypical NF1 deletion generating the chimeric *RNF135-SUZ12* gene maintaining an open reading frame in the transcript, that is abundantly expressed in blood. Interestingly, *RNF135* is a regulator of several tumor suppressor genes [8]. *SUZ12* is a component of the polycomb repressive complex 2 (PRC2) that has an important role during the organism development and, at cellular level, contributes to maintain the cell identity [9]. Furthermore, in this study, data indicating a position effect on expression of genes flanking the deletion have been provided for the first time in NF1 microdeletion syndrome. The patient under clinical follow-up unfortunately currently presents, at 8 years and 5 months old, a severe phenotype with numerous dermal neurofibromas and one plexiform neurofibroma. Given the early worsening of the clinical phenotype, we evaluated a possible expression deregulation of SUZ12 and the other PRC2 components, RNF135 and their target genes, often involved in cancer, to verify their potential role on the early onset of numerous neurofibromas, addressing development of personalized medicine.

## Materials and methods

### Patient recruitment

All clinical data reported were collected during the periodical follow-up of patient 171 [7]. His parents signed an informed consent to study publication and sampling of his biological material.

### Reverse transcription (RT) and quantitative real-time PCR (qPCR)

Total RNA (500 ng), extracted from patient’s and controls’ peripheral blood according to standard procedures, was reverse-transcribed by the Maxima™ H Minus cDNA synthesis master mix with dsDNase (Thermo Fisher Scientific, Waltham, Massachusetts, USA). Two PRC2 components (*EZH2* and *EED*), two RNF135 target genes (*TP53* and *PTEN*), and seven PRC2 target genes (*CDKN2B*, *MECP2*, *PSMD11*, *BCL2*, *CDKN1B*, *NFKB2*, and *EIF3A*), in addition to the *RNF135-SUZ12* and wild-type transcripts, were selected for the expression analysis, where the 2^−ΔCt^ method was applied, using the *TBP* gene as housekeeping control for normalization. The specific oligonucleotides for the qPCR assays are shown in Supplementary Table S1. Each SYBR Green qPCR assay was performed using the GoTaq–qPCR master mix (Promega, Fitchburg, Wisconsin, USA) and run on a QuantStudio 5 Real-Time PCR Systems (Thermo Fisher Scientific).

### Statistical analysis

For each gene analyzed, the mean and the standard deviation were calculated in the patient’s biological triplicate and in the group of healthy controls, which included ten wild-type subjects. The independent samples Student’s *t*-test was applied to compare the means, assuming equal variances, after excluding outliers identified by the Tukey test. The *p*-values were corrected by the Benjamini–Hochberg (BH) method and the results were considered statistically significant when BH-adjusted *p* < 0.05.

## Results

### Clinical description

Patient 171 is male and 8 years 4 months old, previously reported when he was 3 years old [7]. During the following controls, growth was within normal range (see Table 1) [10–12] but he developed multiple cutaneous/subcutaneous neurofibromas, first noticed at 4 years and 3 months old; while at 6 years and 3 months old, a tongue neoformation was noticed, compatible with plexiform neurofibroma for MRI characteristics (see Table 2).

**Table 1:** Growth parameters and percentile

Age	Weight (kg)	Height (cm)	HC (cm)	Reference
Birth	3.42 (50–75° pc)	50.5 (50–75° pc)	34 (50° pc)	[10]
7 m	8.09 (15–50° pc)	71 (50–85° pc)	44 (15–50° pc)	[11]
2 yrs	13 (50° pc)	88 (25–50° pc)	49 (50–85° pc)	[11, 12]
3 yrs 1 m	16.5 (50–75° pc)	100 (50–75° pc)	50 (50–85° pc)	[11, 12]
4 yrs 3 m	17.2 (25–50° pc)	106 (50–75° pc)		[12]
7 yrs 2 m	27.9 (50–75° pc)	128.5 (75° pc)	52	[12]
8 yrs 4 m	33 (75° pc)	135 (75° pc)	52	[12]

*HC*, head circumference; *m*, months; *yrs*, years; *pc*, percentile

**Table 2:** Clinical manifestation

Age	CNF/SCNF (number)	Cutaneous/subcutaneous ultrasound	MRI
4 yrs 3 m	Right wrist		
5 yrs 1 m		Right wrist US: 2 contiguous hypoechoic formations, subcutaneous, at the height of the distal metaepiphyseal region of the radius, of 22 × 7 mm and 6 × 4 mm	
5 yrs 8 m		Frontal skin US: in the subcutis, an oval hypoechoic nodule of about 2.8 × 6 mm and more laterally two other similar contiguous formations, one of 7 × 2 mm and the other of 4 mm	Right Wrist MRI: two subcutaneous small elongated swellings of 20 × 7 mm and 8 × 4 mm on radial profile and a further small fusal area 9 × 3 mm and 7 × 3 mm on the ulnar side; numerous further similar images are observed along the course of the intermuscular vascular-nerve bundles in the forearm
6 yrs 3 m	NF plex of the tongue		
7 yrs 2 m	Frontal (1), neck (1), dorsal paravertebral (1)		Maxillofacial MRI: neoformation compatible for MRI characteristic with plexiform neurofibromas of the body and base on the right of the tongue, extending up to the ipsilateral laryngeal vestibule, with lateral extension to the carotid space and to the right masticatory space and posteriorly to lick the ipsilateral perivertebral space
8 yrs 4 m	Frontal (1), neck (2), back (4), left shoulder (1), left wrist (2), right leg (1)		Cervical spine MRI: cervical spinal roots slightly and diffusely thickened; 10-mm nodularity compatible with NF on the axis of the tooth

*CNF*, cutaneous neurofibroma; *SCNF*, subcutaneous neurofibroma; *m*, months; *yrs*, years

### Gene expression analysis of the chimeric *RNF135-SUZ12* gene and of PRC2 components

**Fig. 1:**
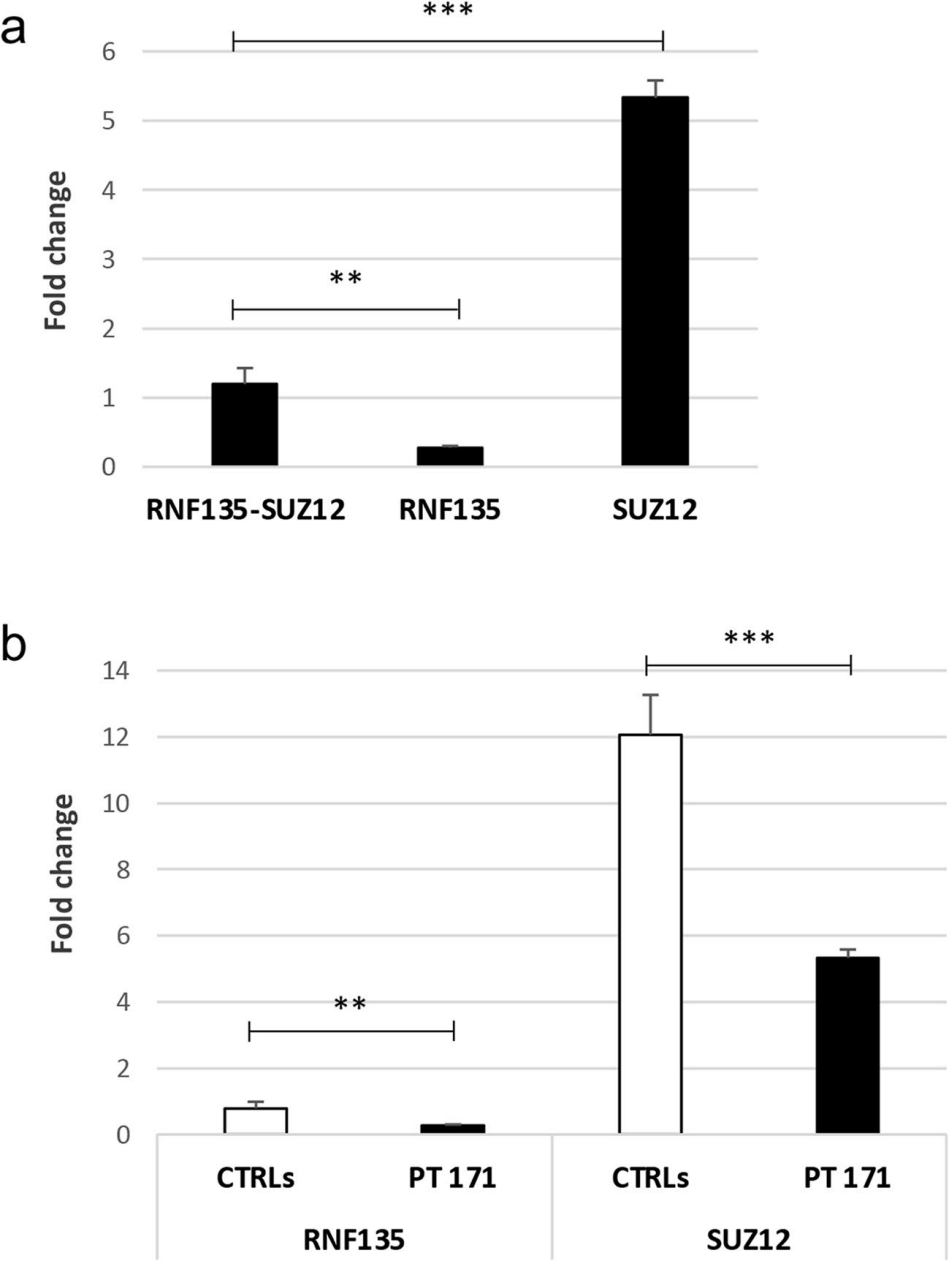
Expression levels of the chimeric RNF135-SUZ12 and wild type transcripts. **a** The chimeric *RNF135-SUZ12* expression level in the patient’s peripheral blood has been compared to that of the wild-type *RNF135* and *SUZ12* transcripts. The expression level of *RNF135-SUZ12* was four times higher than *RNF135* and five times lower than *SUZ12*. **b** The quantitative expression levels of the two wild-type transcripts found in our patient (PT 171) have been compared to the average expression value of ten healthy controls (CTRLs). Compared to controls, the patient showed less than the half of the wild-type transcripts. For the patient, the value is mean ± standard deviation (SD) from three independent biological samples, while for the ten healthy controls the values are means ± SD. **BH-adjusted *p*<0.01, ***BH-adjusted *p*<0.001, Student’s *t*-test

To confirm the presence of the chimeric *RNF135-SUZ12* transcript, already identified in patient 171 when he was 3 years old, and to evaluate its current expression levels, in addition to those of the wild-type *RNF135* and *SUZ12* transcripts, we carried out quantitative RT-PCR assays on the RNA extracted from the patient’s peripheral blood. The qPCR assays revealed, in patient 171, the specific expression of the chimeric *RNF135-SUZ12* transcript, which was expressed four times more than the wild-type *RNF135* transcript and 80% less than the wild-type *SUZ12* transcript (Fig. 1a). The wild-type *RNF135* and chimeric genes share the same 5′UTR; thus, the overexpression of the chimeric gene, compared to the wild-type one, may be caused by an enhancement of the activity of the *RNF135* promoter resulting from the deletion-related position effect. Furthermore, we compared the expression levels of wild-type *RNF135* and *SUZ12* transcripts found in patient 171 to the mean value of those detected in 10 unrelated healthy controls. Patient 171 showed mRNA levels of *RNF135* and *SUZ12* equal to less than the half of the controls (Fig. 1b), consistent with the presence of only one wild-type allele for both the genes.

**Fig. 2:**
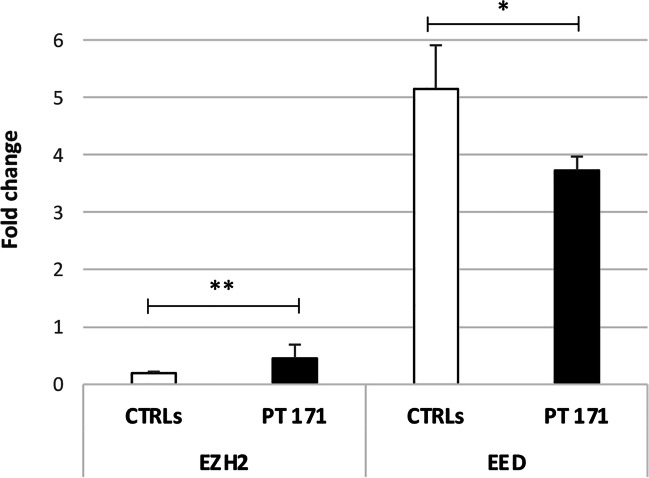
Expression levels of the of the main components of PRC2. *EZH2* and *EED* expression levels in peripheral blood were higher and lower, respectively, in patient 171 (PT 171) compared to ten healthy controls (CTRLs). For the patient, the value is mean ± standard deviation (SD) from three independent biological samples, while for the ten healthy controls the values are means ± SD. *BH-adjusted *p*<0.05, **BH-adjusted *p*<0.01, Student’s *t*-test

Since SUZ12 is part of the PRC2, involved in the transcriptional repression of several genes, often of oncological significance, the gene expression of the other main components of PRC2, named EZH2 and EED, was also evaluated. EZH2, which represents the catalytic subunit of PRC2, is expressed twice in the patient compared to controls (Fig. 2), suggesting an increase in the repressive activity of the complex. EED, which as well as SUZ12 has a regulatory function of stabilizing the structure of the complex, showed a level of expression reduced by half in the patient, compared to the controls (Fig. 2).

### Gene expression analysis of RNF135 and PRC2 target genes

In light of the clinical re-evaluation of the patient 171 phenotype, we assessed the effect of the chimeric gene by performing gene expression analysis of some target genes of RNF135 and PRC2 complex, of which SUZ12 is a component, in order to evaluate possible alterations caused by a gain or loss of function of the chimeric gene, as well as by a reduced level of the wild-type genes.

**Fig. 3:**
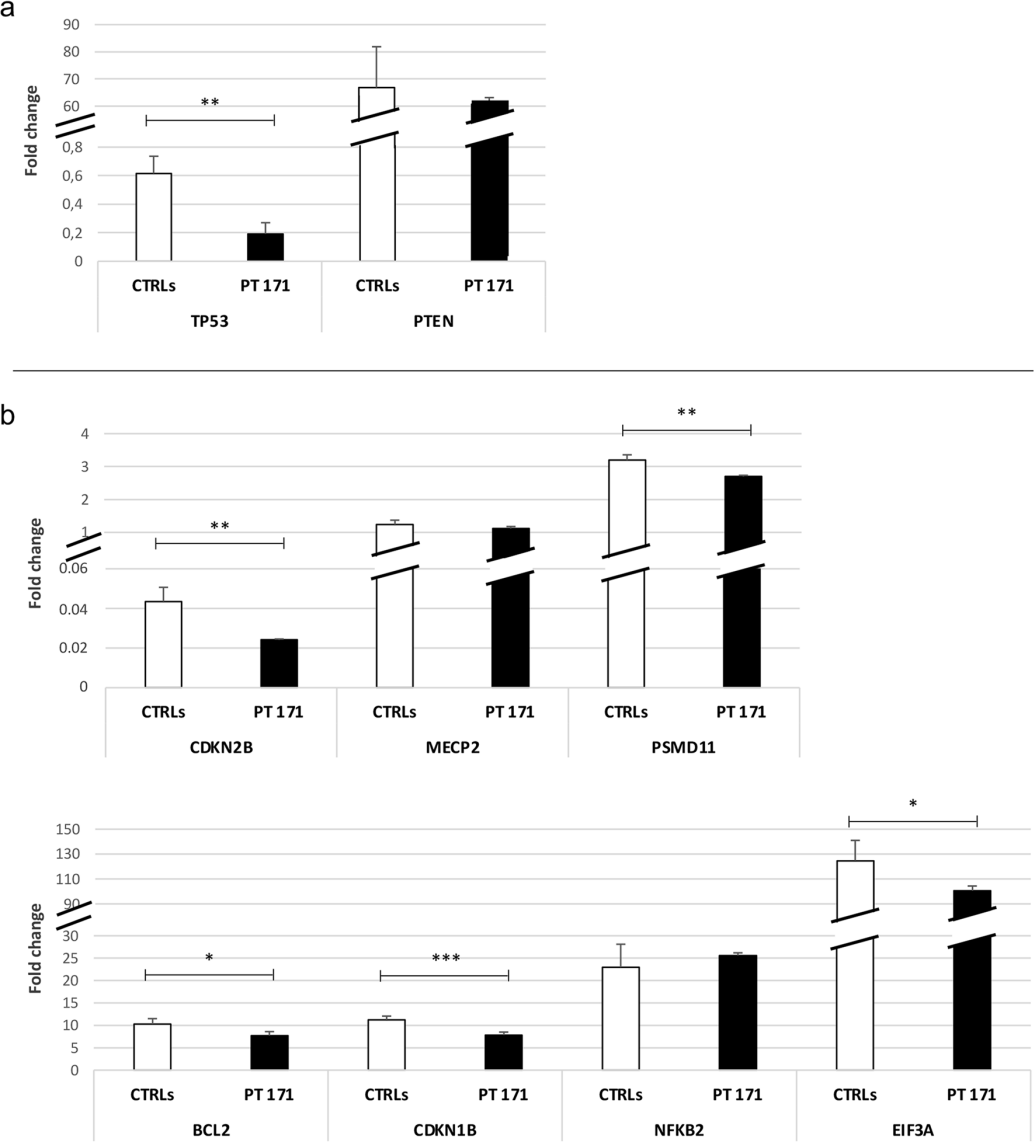
Expression levels of RNF135 and PRC2 target genes. **a** Among the RNF135 targets, the expression level of *TP53* in the patient (PT 171) was one-third of that of the controls (CTRLs). **b** Five out of seven PRC2 target genes analyzed were less expressed in patient 171. The patient’s value is the mean of three independent biological samples ± standard deviation (SD), while the control value represents the average of ten healthy controls ± SD. *BH-adjusted *p*<0.05, **BH-adjusted *p*<0.01, ***BH-adjusted *p*<0.001, Student’s *t*-test

For the analysis of RNF135 target genes, *TP53* and *PTEN* were selected, both tumor suppressor genes whose gene expression is normally significantly promoted by RNF135. While the PTEN expression level was not found to have changed in patient 171, a statistically significant decrease of approximately 70% in the patient’s TP53 expression level was observed compared to healthy controls (Fig. 3a). This suggests that the chimera does not maintain the activity performed by wild-type RNF135. Therefore, the lower expression level of *RNF135* in the patient’s peripheral blood could lead to the decrease of TP53, with consequent reduction of its tumor suppressor activity.

Focusing on PRC2 targets, five out of seven genes analyzed showed a statistically significant decreased expression level in the proband compared to healthy controls. In patient 171, the expression of the genes *CDKN2B*, *CDKN1B*, *BCL2*, *EIF3A*, and *PSMD11* is reduced by 45%, 30%, 25%, 20%, and 15%, respectively (Fig. 3b). For *MECP2* and *NFKB2* no significant changes in expression level were found.

Based on the decrease in the expression levels of *SUZ12* transcript in patient 171, an increased expression of its target genes was expected, which instead was shown to be reduced in the patient, suggesting a higher transcriptional repression mediated by the PRC2 complex. The reduced expression levels of the *TP53* gene and some PRC2 targets, often of oncological significance, are in line with the early development, in prepubertal age, of a large number of neurofibromas in the patient 171.

## Discussion

We re-evaluated our patient 171, highlighting a clinical worsening, characterized by a heavier neurofibroma burden compared to the available data on atypical deletions; subsequently, we studied the impact of the chimeric gene *RNF135-SUZ12*, generated by the 17q11.2 deletion carried by the patient, on the targets of the two partner genes and finally on the clinical phenotype.

Whereas the onset of cutaneous and subcutaneous neurofibromas is usually seen after 8–10 years of age in patients with intragenic *NF1* mutations, and in microdeletion type 1 NF1 individuals an earlier onset is not uncommon, it is difficult to define the neurofibromas burden in patients with atypical deletion [3]. Moreover, since the extreme clinical variability and the lack of detailed clinical description, it is difficult to compare the clinical features in our patient, sex- and age-matched, with the other atypical microdeletion cases reported. To the best of our knowledge, only 61 atypical NF1 microdeletions have been reported so far, of which 31 extending beyond type 1 NF1 microdeletion breakpoints and 30 smaller deletions with breakpoints located within the type 1 microdeletion region. Details of clinical phenotype are available only for 9/30 patients with small atypical deletions [13]. Patient 310221, reported by Kehrer-Sawatski et al., had just a small cutaneous neurofibroma on his chest at 6 years old [13]. The presence of cutaneous neurofibromas was also described in patients NF056 and NF073 reported by Zhang et al., with onset at 26 and 8 years old respectively [14].

The patient at age 3 expressed the chimeric transcript *RNF135-SUZ12* that was predicted to maintain the open reading frame [7]. Because a large part of *RNF135* is not present, presumably its physiological function is not maintained in the chimeric gene. Since the chimeric gene maintains most of the *SUZ12* functional domains, it could have acquired a new function that could modify the activity of the PRC2, a repressive complex of which *SUZ12* represents a stabilizing subunit.

Because both *RNF135* and *SUZ12* encode regulatory factors involved in gene expression [8, 15], we have extended the analysis to the target genes of *RNF135* and *PRC2*, to possibly infer the role of the chimeric gene. The chimeric transcript is currently expressed by the patients. The significant and tendential gene expression reduction of tumor suppressors *TP53* and *PTEN* in patient 171, normally induced by *RNF135* [8], is consistent with the reduced wild-type transcript and strongly indicates that the chimeric gene loses the normal function of *RNF135*. Instead, 5 out of 7 PRC2 target genes analyzed show a statistically significant decrease in expression levels, in patient 171 compared to healthy controls, highlighting an increased activation of the PRC2 repressive complex, that led us to hypothesize a gain-of-function modification of the RNF135-SUZ12 chimera.

**Fig. 4:**
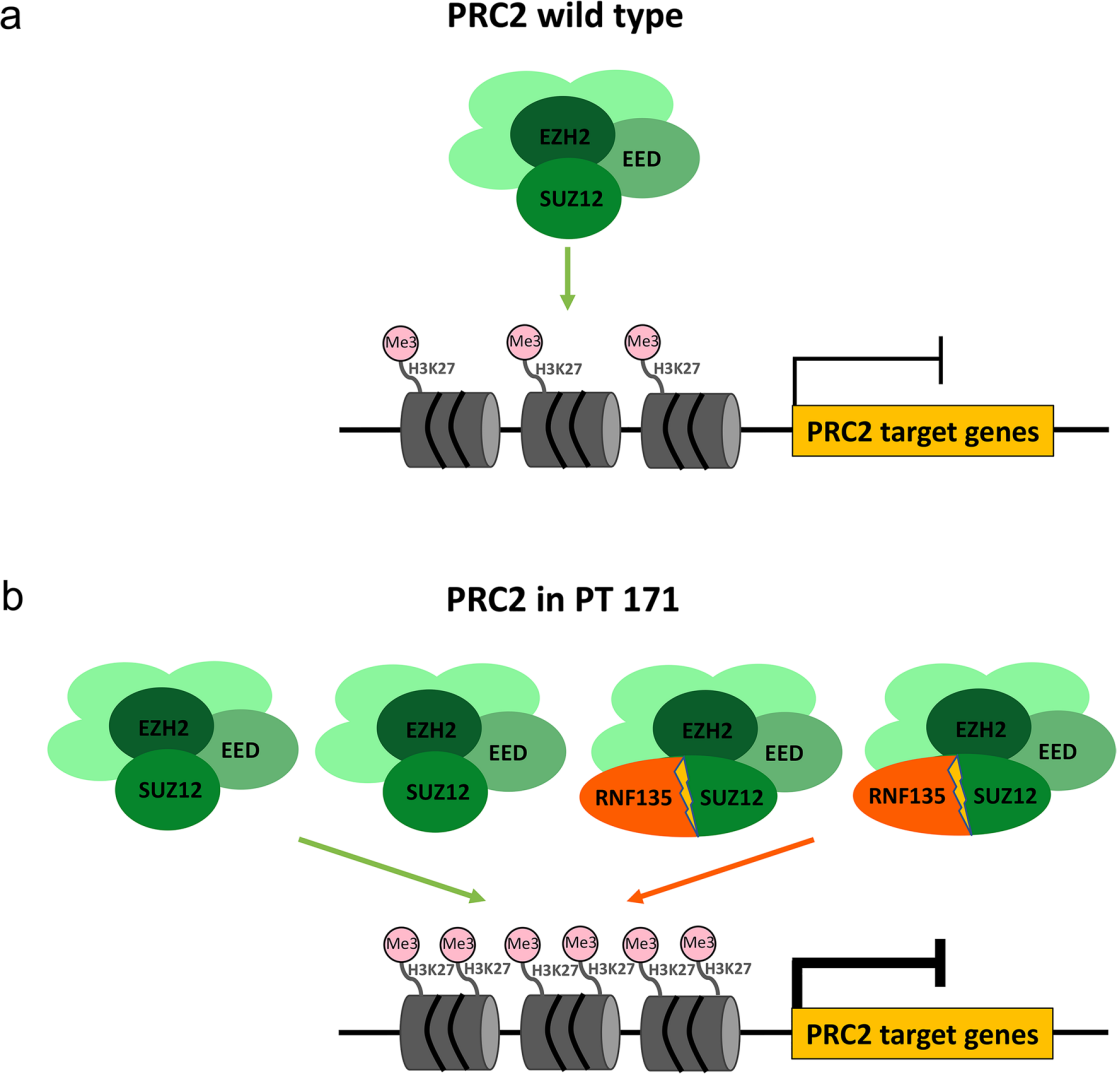
Activity of polycomb repressive complex 2, in wild-type subjects and in patient 171. **a** The wild-type PRC2 complex, composed by the core subunits (EZH2, SUZ12, and EED) and other accessory subunits (shown in light green), represses the transcriptional expression of several target genes through histone H3 lysine 27 (H3K27) trimethylation (Me3) of their promoters. **b** In our patient (PT 171), reduced expression of PRC2 target genes suggests increased activity of the complex. We hypothesize a gain of function of the chimeric gene *RNF135-SUZ12*, which could stabilize the complex more, and a greater aggregation of the PRC2 complex, resulting from the increase of the catalytic subunit EZH2

We speculate that the RNF135-SUZ12 chimeric product could play a role in the increased repressive activity of PRC2 by increasing the stability of the complex. Consistently, the chimeric gene of patient 171 retains most of the functional domains of SUZ12, whose role is to stabilize the PRC2 complex. Furthermore, the chimeric gene showed, compared to wild-type *SUZ12*, an additional zinc finger domain (aa 21–63), which derives from the *RNF135* portion retained by the chimera that probably acquire an increased stabilizing function, thereby contributing to the expression deregulation of PRC2 target genes (Fig. 4).

The hyperactivation of PRC2 complex could also be enhanced by the increased expression of the catalytic subunit of the complex, *EZH2*, which was detected in the patient’s peripheral blood. We hypothesize that the increase in EZH2 subunit could promote the aggregation of a higher number of PRC2 complexes (Fig. 4). *EZH2* gene is regulated by a transcription factor, named MYCN [16], a protooncogene that has been described to assume an oncogenic function when amplified. Of note, *MYCN* amplification has been correlated with an increase in Akt phosphorylation, given that MYCN is in turn regulated by the PIK3/Akt signaling pathway [17], which is known to be induced by hyperactivation of the RAS pathway, typically present in NF1 patients. Therefore, the hyperactivation state of the RAS pathway could indirectly induce the overexpression of *EZH2*, which was detected in patient 171 and which could contribute to the pathogenesis of NF1 patients. Interestingly, the *EZH2* overexpression is correlated with a negative prognosis and short survival in different types of tumors [18]. Therefore, the increase in EZH2 could play a role in the cancer predisposition of NF1 patients.

When SUZ12 is lost, EZH2 acquires an anomalous function, going to carry out its activity through a non-canonical pathway for PRC2 [19], while in our patient we found a reduced expression of the PRC2 targets, which suggests that the complex is more active, and which may make the patient susceptible to the onset of a high number of neurofibromas in relation to his age. Further studies will be carried out to understand if the chimeric product is able to interact with the complex, enhancing its activity.

## Conclusions

Atypical NF1 microdeletions are of particular interest since they could help in understanding the physiopathological mechanism underlying the manifestation in this condition and define a genotype-phenotype correlation.

In particular, the obtained results, providing new insights on the alterations caused by the described genetic lesion, may open new perspectives on the elucidation of regulatory mechanisms of PRC2 complex. Given that the patients with NF1 type microdeletion syndrome, frequently showing the lost or disruption of *SUZ12*, are more susceptible to tumor development, further studies on selected and larger cohorts of NF1 microdeletion patients, with a severe tumor phenotype, could shed light on the role of the members of PRC2 complex in tumor susceptibility in neurofibromatosis type 1, allowing the identification of therapeutic targets that can contribute to effective treatment.

## Supplementary information

ESM 1 (XLSX 10 kb)

## Data Availability

The data analyzed during the current study are available from the corresponding author on reasonable request.
